# Current Status of Adverse Event Profile of Cyclosporine in Kidney, Stem Cell, and Heart Transplantations Using the Japanese Pharmacovigilance Database

**DOI:** 10.7759/cureus.29383

**Published:** 2022-09-20

**Authors:** Iku Niinomi, Saki Oyama, Ayaka Inada, Tomohito Wakabayashi, Tatsuya Iida, Hiroko Kambara, Mayako Uchida, Yukako Sano, Keiko Hosohata

**Affiliations:** 1 Education and Research Center for Clinical Pharmacy, Osaka Medical and Pharmaceutical University, Takatsuki, JPN; 2 Education and Research Center for Pharmacy Practice, Doshisha Women's College of Liberal Arts, Kyotanabe-shi, JPN

**Keywords:** japanese adverse drug event report (jader) database, reporting odds ratio (ror), spontaneous reporting system, transplantation, cyclosporine

## Abstract

Background: Cyclosporine is widely used to prevent allograft rejection after transplantation. The purpose of this study was to clarify the adverse events profiles associated with cyclosporine in transplant patients using a spontaneous reporting system database.

Methods: Retrospective pharmacovigilance disproportionality analysis was conducted using the Japanese Adverse Drug Event Report (JADER) database, with the reporting odds ratio (ROR) and 95% confidence interval (CI) for each adverse event.

Results: The database comprised 3,327, 958, and 956 reports associated with cyclosporine in the kidney, stem cell, and heart transplant patients, respectively. Infectious and renal disorders were commonly detected in these transplant patients. The signal scores of cyclosporine for toxic nephropathy were noteworthy in the kidney (ROR: 15.1, 95% CI: 11-20.8) and stem cell (ROR, 216; 95% CI, 29.3-1593) transplantation. Cyclosporine in heart transplantation was strongly associated with gastric cancer (ROR, 39.4; 95% CI, 16.7-93.2), but not kidney or stem cell transplantation.

Conclusion: It was suggested that there is a diversity in the strength of the association between cyclosporine and adverse events in the kidney, stem cell, and heart transplantation. Our results may provide useful information for treatment with cyclosporine, although further research with more data is needed.

## Introduction

Calcineurin inhibitors, immunosuppressive agents, have been used for decades in solid organ transplantation to prevent rejection with improvement in long-term survival [1]. Cyclosporine has a similar mechanism of effect to tacrolimus, but experience with cyclosporine is much more extensive than that with tacrolimus. Cyclosporine as well as tacrolimus are narrow therapeutic index medications, which exhibit marked inter- and intra-patient variabilities on systemic exposure [2]. Therapeutic drug monitoring of cyclosporine in transplant patients has markedly evolved in routine clinical practice for individualized medicine.

Cyclosporine causes a large spectrum of adverse effects, and several adverse events have been reported, such as nephrotoxicity, arterial hypertension, neurotoxicity, increased rate of infections, diabetes, and cardiomyopathy [3, 4]. However, there is little information about adverse events that are most frequently responsible for cyclosporine used in all kinds of transplantation in a real-world setting.

Recently, spontaneous reporting systems have been utilized as a useful method of post-marketing drug safety surveillance for the detection of new adverse drug events or changes in the occurrence of adverse events, referred to as signal detection [5, 6]. The Japanese Adverse Drug Event Report (JADER) database is a largely published database for pharmacovigilance [7-10]. The objective of this study was to clarify the profile of adverse events by cyclosporine used in a variety of transplant patients using the JADER database.

## Materials and methods

The JADER dataset is publicly available and can be downloaded from the website of the Pharmaceuticals and Medical Devices Agency (PMDA) (www.pmda.go.jp). We used the dataset to which adverse event reports were submitted between April 2004 and January 2017. The JADER consists of the following types of data: patient demographic information (DEMO), drug information (DRUG), adverse events (REAC), and medical history. The DEMO table was then linked to the DRUG and REAC tables using the ID number, as reported previously [7,9,11]. The contribution of the medication to adverse events was classified into three categories: “suspected medicine,” “concomitant medicine,” and “interaction.” A “suspected medicine” is defined as a pharmaceutical product suspected of causing an adverse event. When the reporter suspects an interaction, he/she reports it as an “interaction.” A “concomitant medicine” is defined as another pharmaceutical product used at the time of the adverse reaction. In signal detection analysis, a masking effect is defined as a condition whereby a given drug-event pair might be hidden by the presence of another product [12]. Then, we only extracted cases that were classified as “suspected medicine.”

**Figure 1:**
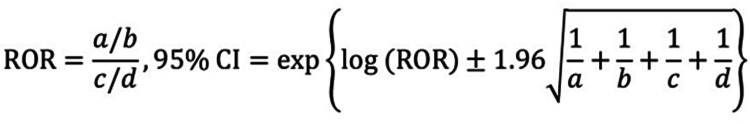
ROR is calculated by the formula where a - the number of patients with a target event when they received a target drug, b - the number of patients with non-target adverse events when they received a target drug, c - the number of patients with the target event when they received non-target drugs, d - the number of patients with non-target adverse events when they received non-target drugs

The adverse events and indications in the JADER database are coded as Preferred Terms (PTs), each of which is a single medical concept for a symptom, sign, disease diagnosis, therapeutic indication, or investigation, using the Medical Dictionary for Regulatory Activities (MedDRA). We compiled a cross-tabulation table based on two classifications: the presence or absence of the adverse event, and the suspected medicine. Then, we calculated the reporting odds ratio (ROR) by the following formula (Figure 1).

Generally, ROR is used with the spontaneous reporting database as an index of the relative risk of drug-associated adverse events. A signal was considered to be present when the lower limit of the 95% confidence intervals (CI) of the ROR was > 1.

In this database, age, height, and weight information are indicated as follows: age in decades, height in centimetres, and weight in kilograms. These data are not given as continuous variables because of privacy considerations, so we could not conduct multiple analyses using them. All analyses were conducted using JMP Pro 12 (SAS Institute Inc., Cary, NC, USA).

## Results

The database comprised 20408 adverse event reports associated with cyclosporine. Of 20,408, 3,327 (16.3%), 1,499 (7.3%), 1,102 (5.4%), 958 (4.7%), and 956 (4.7%) reports involved kidney transplantation, nephrotic syndrome, aplastic anemia, stem cell transplantation, and heart transplantation, respectively (Table 1).

**Table 1:** Most frequent reasons for use of cyclosporine

	N (%)
Renal transplant	3,327 (16.3)
Nephrotic syndrome	1,499 (7.3)
Aplastic anemia	1,102 (5.4)
Stem cell transplant	958 (4.7)
Heart transplant	956 (4.7)
Psoriasis	930 (4.6)
Prophylaxis against transplant rejection	921 (4.5)
Unknown/Others	10,715 (52.5)

To gain insight into how cyclosporine is associated with adverse events under its use for the prevention of rejection after transplantation, we focused on kidney, stem cell, and heart transplantations for further analysis. As shown in Table 2, approximately 60%, 50%, and 90% of patients were male in the kidney, stem cell, and heart transplant patients with adverse events receiving cyclosporine, respectively. According to the age distribution of these transplant patients, adverse events associated with cyclosporine occurred frequently in kidney transplant patients in their 50s, stem cell transplant patients in their 50s, and heart transplant patients in their 10s, respectively. The mean dose of cyclosporine was 201.6 mg, 96.8 mg, and 149.5 mg in kidney transplant patients, stem cell transplant patients, and heart transplant patients, respectively.

**Table 2:** Characteristics of the patients with adverse events associated with cyclosporine in the kidney, stem cell, and heart transplantation Values are expressed as number (%) or mean ± SD.

Variable		n (%) / mean ± SD				
		Kidney transplantation		Stem cell transplantation		Heart transplantation
Sex						
	Men	2058 (61.9)		474 (49.5)		839 (87.8)
	Women	1247 (37.5)		453 (47.3)		115 (12.0)
	Unknown	22 (0.7)		31 (3.2)		2 (0.2)
Age						
	Under 10	76 (2.3)		17 (1.8)		155 (16.2)
	10s	140 (4.2)		87 (9.1)		237 (24.8)
	20s	402 (12.1)		105 (11.0)		64 (6.7)
	30s	615 (18.5)		114 (11.9)		23 (2.4)
	40s	596 (17.9)		256 (26.7)		162 (16.9)
	50s	733 (22.0)		318 (33.2)		229 (24.0)
	60s	573 (17.2)		37 (3.9)		81 (8.5)
	≥70s	72 (2.2)		0 (0)		0 (0)
	Unknown	120 (3.6)		24 (2.5)		5 (0.5)
Dose of cyclosporine, mg		201.6 ± 139.9		96.8 ± 81.5		149.5 ± 93.0

In these transplantations, infectious and renal adverse events were common. Especially, Cytomegalovirus infection was highly ranked and significantly correlated with cyclosporine in these transplant patients. As shown in Table 3, the top 10 adverse events associated with cyclosporine in kidney transplantation are listed and we evaluated each adverse event using ROR and 95% CI. In Table 3, cytomegalovirus infection (358 reports), urinary tract infection (179 reports), toxic nephropathy (147 reports), and pneumonia (96 reports) ranked highly. Of note, the association of cyclosporine with toxic nephropathy was noteworthy (ROR, 15.1; 95% CI, 11-20.8).

**Table 3:** The top 10 adverse events associated with cyclosporine in kidney transplantation *Signal detected. CI, confidence interval; PT, preferred terms; ROR, reporting odds ratio.

PT	n	ROR	95%CI
Cytomegalovirus infection	358	1.37	1.21-1.55*
Urinary tract infection	179	3.9	3.2-4.76*
Nephropathy toxic	147	15.1	11-20.8*
Pneumonia	96	1.94	1.53-2.46*
Diabetes mellitus	77	2.12	1.62-2.77*
Hypertension	75	3.65	2.71-4.92*
Blood creatinine increased	60	1.2	0.9-1.6
Renal impairment	60	1.43	1.07-1.91*
Escherichia urinary tract infection	60	2.62	1.91-3.58*
Pneumocystis jirovecii pneumonia	58	1.61	1.19-2.17*

Table 4 shows the 10 most frequently reported adverse events by cyclosporine in stem cell transplantation. The most frequently reported adverse event was thrombotic microangiopathy (TMA) (51 reports), and its ROR was 3.83 (95% CI, 2.73-5.37). The signal score of adverse events caused by cyclosporine in stem cell transplantation for toxic nephropathy was highest (ROR, 216; 95% CI, 29.3-1593), followed by membranous glomerulonephritis (ROR, 190; 95% CI, 25.7-1412), and nephrotic syndrome (ROR, 95.2; 95% CI, 22.4-404).

**Table 4:** The top 10 adverse events associated with cyclosporine in stem cell transplantation *Signal detected. CI, confidence interval; PT, preferred terms; ROR, reporting odds ratio.

PT	n	ROR	95%CI
Thrombotic microangiopathy	51	3.83	2.73-5.37*
Renal disorder	47	10.5	6.7816.1*
Cytomegalovirus infection	27	2.64	1.7-4.1*
Nephropathy toxic	26	216	29.3-1593*
Nephrotic syndrome	23	95.2	22.4-404*
Glomerulonephritis membranous	23	190	25.7-1412*
Liver disorder	22	1.21	0.77-1.91
Neutropenia	22	1.65	1.04-2.62*
Pneumocystis jirovecii pneumonia	21	17.3	8.13-36.9*
Staphylococcal sepsis	21	1.84	1.14-2.97*

Unlike the adverse event profiles of cyclosporine in kidney and stem cell transplantation, the associations of cyclosporine with gastric cancer (ROR, 39.4; 95% CI, 16.7-93.2), pneumonia pneumococcal (ROR, 11.4; 95% CI, 6.24-20.9), and cholecystitis (ROR, 11.4; 95% CI, 6.24-20.9) were noteworthy in heart transplantation (Table 5).

**Table 5:** The top 10 adverse events associated with cyclosporine in heart transplantation *Signal detected. CI, confidence interval; PT, preferred terms; ROR, reporting odds ratio.

PT	n	ROR	95%CI
Tonsillitis	90	8.48	6.11-11.8*
Pharyngitis	48	2.28	1.62-3.21*
Renal impairment	42	1.41	1-1.99*
Gastric cancer	41	39.4	16.7-93.2*
Pyrexia	36	2.12	1.43-3.12*
Cholelithiasis	32	10.7	5.94-19.4*
Pneumonia pneumococcal	32	11.4	6.24-20.9*
Cholecystitis	32	11.4	6.24-20.9*
Blood creatine phosphokinase increase	32	10.1	5.67-18.1*
Cytomegalovirus infection	31	4.63	2.87-7.48*

## Discussion

Using a large and nationwide study of pharmacovigilance data, we conducted a comparison of safety profiles by cyclosporine among kidney, stem cell, and heart transplant patients. In this study, renal and infectious adverse events were commonly detected in these transplant patients. Especially, cytomegalovirus infection was highly ranked and significantly correlated with cyclosporine in these transplant patients. On the other hand, there was variability in the safety profile of cyclosporine among these transplantations: cyclosporine in heart transplantation was significantly correlated with gastric cancer, but not in kidney or stem cell transplantation; however, cyclosporine in kidney transplantation was significantly correlated with toxic nephropathy, and cyclosporine in stem cell transplantation was significantly correlated with toxic nephropathy, nephrotic syndrome, and membranous glomerulonephritis. To the best of our knowledge, this is the first study to clarify the profiles of adverse events by cyclosporine in the kidney, stem cell, and heart transplant patients using a spontaneous reporting database.

Our pooled analysis of various types of transplantation showed that infectious adverse events were commonly reported: pneumonia pneumococcal and Cytomegalovirus infection in heart transplantation, Cytomegalovirus infection, urinary tract infection, Escherichia urinary tract infection, and Pneumocystis jirovecii pneumonia in kidney transplantation, and Cytomegalovirus infection, Pneumocystis jirovecii pneumonia, and staphylococcal sepsis in stem cell transplantation. Transplant patients by immunosuppressant use are at higher risk of infection compared with the general population. It has been reported that abnormalities in thymi and T-cell generation after cyclosporine administration are observed [13]. For example, cyclosporine causes involution of the thymic medulla [13], decreased single-positive thymocytes and peripheral T cells, and downregulated expression of class II MHC [14]. Then, abnormalities in thymi and T-cell generation in the presence of calcineurin inhibitors may provide an explanation for the increased risk of infection.

As for adverse events by cyclosporine in kidney transplantation, the signal score of cyclosporine was significantly higher for toxic nephropathy (ROR, 15.1; 95% CI, 11-20.8), urinary tract infection (ROR, 3.9; 95% CI, 3.2-4.76), and hypertension (ROR, 3.65; 95% CI, 2.71-4.92). Several studies have reported that cyclosporine was nephrotoxic in humans [15,16]. Patients treated chronically with cyclosporine showed that the decreases in glomerular filtration and renal perfusion were accompanied by a reduced proximal reabsorptive capacity [16]. In another study, activation of the vasoconstriction systems contributed to cyclosporine-induced nephrotoxicity [17]. In addition, cyclosporine elevated angiotensin II levels, angiotensin-converting enzyme activity, and angiotensin II type I receptor expression, which augments angiotensin-induced vasoconstriction [18,19]. The renal vasoconstriction can cause tissue hypoxia and enhance the formation of reactive oxygen species (ROS) that ultimately cause cellular injury and apoptosis. ROS plays an important role in cyclosporine-induced nephrotoxicity [20,21]. Cyclosporine-mediated induction of oxidative stress is associated with cyclophilin D, with cyclophilin D deletion providing a protective effect [22]. In our results, cyclosporine was significantly correlated with hypertension. It is consistent with a report that calcineurin inhibitor exposure is associated with irreversible changes in blood vessels (arterial hyalinosis) [18]. As another mechanism, underlying hypertension by cyclosporine, cyclosporine-mediated water, and sodium retention was demonstrated to contribute to the progression of hypertension in animal models [23].

As for adverse events by cyclosporine in stem cell transplantation, our post-marketing data revealed that the signal scores for toxic nephropathy, nephrotic syndrome, and membranous glomerulonephritis were noteworthy. It is consistent with a clinical study in adult patients receiving cord blood transplantation at significant acute kidney injury (AKI) risk, and AKI is associated with an increased risk of chronic kidney disease [24]. The mechanism of cyclosporine-induced nephrotoxicity is partly due to total cyclosporine exposure [25]. On the other hand, no correlation has been reported between blood cyclosporine levels and the incidence of renal failure in allogeneic hematopoietic cell transplantation [26]. Of note, our results showed that TMA was correlated with cyclosporine in stem cell transplantation. Transplant-associated TMA is multifactorial, and the risk factors include therapy used in conditioning regimens, human leukocyte antigen mismatch, graft-versus-host disease (GVHD), and viral infections, exposure to calcineurin inhibitors s, although its pathophysiology is poorly understood. One report showed that patients with transplantation-associated TMA were only identified during or after cyclosporine immunosuppression [27]; whereas another report showed that the use of cyclosporine /tacrolimus-based GVHD prophylaxis was not a risk factor for transplant-associated-TMA in pediatric patients with hematopoietic stem cell transplantation [28].

As for adverse events by cyclosporine in heart transplantation, gastric cancer showed a positive association with cyclosporine in heart transplantation, and its signal score was noteworthy (ROR, 39.4; 95% CI, 16.7-93.2). Our results are consistent with the reports that the incidence of cancer is high in the transplant population [29-31]. Immunosuppressive drugs have been recognized as a major factor contributing to the increased incidence of cancer [29,30], especially virus-related cancers, in transplant recipients, suggesting that the increase is due to the loss of immune control of oncogenic viruses [29]. The incidence of malignancies might be related to the type of allograft; as reported previously, the highest incident rate of skin cancer was observed in heart transplant recipients and the lowest in liver transplant recipients [32]. This marked discrepancy in cancer frequency is partly due to the more intense immunosuppression used for the prevention of allograft rejection in organs. Cyclosporine treatment regimens in Japan are 9-12 mg/kg per day, 6-12 mg/kg per day, and 10-15 mg/kg per day in the kidney, stem cell, and heart transplant patients, respectively. Our results showed that doses of cyclosporine were highest for kidney transplant patients, followed by heart transplant patients and stem cell transplant patients. However, their age distributions were different; teenagers are most common among heart transplant patients, unlike kidney and heart transplant patients. Generally, teenage body weights are lower than in middle and old age, leading to lower doses in heart transplant patients than in kidney transplant patients. Then, the development of gastric cancer when cyclosporine was used for heart transplantation could be partly due to the cyclosporine treatment protocol after heart transplantation. In this study, the use of cyclosporine in heart transplantation showed a positive signal with gastric cancer, which will provide important information in clinical settings. 

This pharmacovigilance study using the JADER database has several limitations. First, as in all pharmacovigilance studies, we were unable to calculate the true incidence rates, because of a lack of the total number of patients receiving the drugs of interest and underreporting. Especially, adverse events that are well-known by certain drugs are less likely to be reported. Second, in spontaneous reporting systems such as JADER, control populations are not included, so ROR is different from the “odds ratio” which is often used in epidemiological studies. In real terms, ROR denotes an increased likelihood of reporting an adverse event and not the risk of an adverse event per se. Third, the extent of actual exposure in the treated population is not available from the database. Finally, there might be other confounding factors associated with the adverse events, but the present method did not provide us with detailed clinical information on the patients.

## Conclusions

This is the first study to reveal that there is diversity in the strength of association between cyclosporine and adverse events in kidney, stem cell, and heart transplantation in a real-world setting. This diversity is partly due to the age of the patients who develop the complication after immunosuppression and the cyclosporine treatment protocol for the patients after these transplantations. Physicians should be alerted in order to take precautions against the associated adverse events, and so potentially avoid them.
